# Anaplastic Lymphoma Kinase Is a Regulator of Alcohol Consumption and Excitatory Synaptic Plasticity in the Nucleus Accumbens Shell

**DOI:** 10.3389/fphar.2017.00533

**Published:** 2017-08-15

**Authors:** Regina A. Mangieri, Esther Y. Maier, Tavanna R. Buske, Amy W. Lasek, Richard A. Morrisett

**Affiliations:** ^1^Division of Pharmacology and Toxicology, College of Pharmacy, The University of Texas at Austin, Austin TX, United States; ^2^Department of Psychiatry, University of Illinois at Chicago, Chicago IL, United States

**Keywords:** electrophysiology, operant self-administration, glutamate, two-bottle choice, mice

## Abstract

Anaplastic lymphoma kinase (ALK) is a receptor tyrosine kinase recently implicated in biochemical, physiological, and behavioral responses to ethanol. Thus, manipulation of ALK signaling may represent a novel approach to treating alcohol use disorder (AUD). Ethanol induces adaptations in glutamatergic synapses onto nucleus accumbens shell (NAcSh) medium spiny neurons (MSNs), and putative targets for treating AUD may be validated for further development by assessing how their manipulation modulates accumbal glutamatergic synaptic transmission and plasticity. Here, we report that *Alk* knockout (*Alk*^KO^) mice consumed greater doses of ethanol, relative to wild-type (*Alk*^WT^) mice, in an operant self-administration model. Using *ex vivo* electrophysiology to examine excitatory synaptic transmission and plasticity at NAcSh MSNs that express dopamine D1 receptors (D1MSNs), we found that the amplitude of spontaneous excitatory post-synaptic currents (EPSCs) in NAcSh D1MSNs was elevated in *Alk*^KO^ mice and in the presence of an ALK inhibitor, TAE684. Furthermore, when ALK was absent or inhibited, glutamatergic synaptic plasticity – long-term depression of evoked EPSCs – in D1MSNs was attenuated. Thus, loss of ALK activity in mice is associated with elevated ethanol consumption and enhanced excitatory transmission in NAcSh D1MSNs. These findings add to the mounting evidence of a relationship between excitatory synaptic transmission onto NAcSh D1MSNs and ethanol consumption, point toward ALK as one important molecular mediator of this interaction, and further validate ALK as a target for therapeutic intervention in the treatment of AUD.

## Introduction

One major objective of neuropsychopharmacological research is the identification and validation of novel targets for medication developments for substance use disorders (SUDs) – especially prevalent SUDs such as alcohol use disorder (AUD). In recent years, the receptor tyrosine kinase anaplastic lymphoma kinase (ALK) has been implicated as a modulator of biochemical, physiological, and behavioral responses to ethanol and psychostimulants (Lasek et al., 2011a,b; He et al., 2015; Schweitzer et al., 2016; Dutton et al., 2017). In this report, we further investigate mechanisms through which ALK might be validated as a target for AUD medication development.

Anaplastic lymphoma kinase was first identified as a potential target for treating SUDs by Lasek and colleagues’ discovery that ALK regulates behavioral responses to cocaine. Pretreatment of mice with an ALK inhibitor reduced cocaine-conditioned place preference, and attenuated locomotor sensitization to repeated cocaine injections – an effect recapitulated by knockdown of *Alk* expression in the nucleus accumbens (NAc) (Lasek et al., 2011a). Subsequent work by Lasek colleagues then implicated *Alk* in responses to ethanol as well. Genetic deletion of *Alk* altered ethanol-induced sedation of fruit flies and mice, and increased consumption in a mouse model of ethanol binge-drinking (Lasek et al., 2011b). More recently, Schweitzer et al. (2016) reported a similar finding, whereby *Alk* knockout mice (*Alk*^KO^) drank more ethanol than wild-type mice (*Alk*^WT^) during two-bottle choice (ethanol vs. water) testing. Strikingly, however, *Alk*^KO^ did not show the escalation in two-bottle choice ethanol drinking that normally results from repeated cycles of chronic, intermittent, ethanol (CIE) vapor exposure and withdrawal. This pattern of effects was paralleled in spontaneous gamma-aminobutyric acid (GABA) transmission in the central nucleus of the amygdala (CeA): ethanol naïve *Alk*^KO^ exhibited greater frequency of GABAergic inhibitory post-synaptic current (IPSC) events relative to *Alk*^WT^, but CIE did not increase IPSC frequency in *Alk*^KO^ as it did in *Alk*^WT^. Additionally, Lasek’s group has shown that ALK signaling is activated by ethanol and ALK inhibition attenuates ethanol-induced ERK phosphorylation (He et al., 2015). Importantly, the evidence of a relationship between ALK and ethanol responses extends to humans. Single nucleotide polymorphisms in human *ALK* were associated with behavioral sensitivity to ethanol in an experimental setting (Lasek et al., 2011b) and alcohol dependence in a meta-analysis of genome-wide association studies (Wang et al., 2011).

Although ALK was originally identified because of its role in non-Hodgkin’s, anaplastic large cell lymphoma, it is primarily expressed in neural tissue (Morris et al., 1994; Iwahara et al., 1997; Hurley et al., 2006; Vernersson et al., 2006), and has been shown to play critical roles in nervous system development and function (Liao et al., 2004; Rohrbough and Broadie, 2010; Yao et al., 2013). In particular, ALK appears to regulate synaptic architecture and transmission strength (Rohrbough et al., 2013; Schweitzer et al., 2016), suggesting a general mechanism by which ALK manipulations might impact responses to psychostimulants and ethanol. Thus, despite its field of discovery, the body of work implicating ALK in neuropsychopharmacology, especially in relation to psychostimulant- and ethanol-reinforced behaviors, prompts further study of this target for potential novel medication development.

One consistent feature of neuroadaptations elicited by repeated psychostimulant or ethanol exposure is altered alpha-amino-3-hydroxy-5-methyl-4-isoxazole propionic acid receptor (AMPAR)- and *N*-methyl-D-aspartate receptor (NMDAR)-mediated transmission at corticolimbic synapses on NAc medium spiny neurons (MSNs) (Jeanes et al., 2011, 2014; Grueter et al., 2012; Marty and Spigelman, 2012; Abrahao et al., 2013; Spiga et al., 2014; Renteria et al., 2016b, 2017; Ji et al., 2017). Indeed, we recently proposed that novel targets for the treatment of AUD might be validated for their potential therapeutic development by assessing how their manipulation modulates NMDAR-dependent long-term depression (LTD) of AMPAR-mediated excitatory post-synaptic currents (EPSCs) in NAc shell (NAcSh) MSNs (Renteria et al., 2016a).

Herein, we provide new evidence in support of this approach to target validation. We began by comparing *Alk*^KO^ and *Alk*^WT^ mice in an operant self-administration paradigm to verify the effect of *Alk* deletion on ethanol-reinforced behaviors in our hands. We then examined the effects of *Alk* deletion or acute pharmacological inhibition of ALK on the physiology of D1 dopamine receptor-expressing MSNs (D1MSNs) in the NAcSh, and focused subsequent investigation on glutamatergic input to these neurons since our prior and current work particularly implicates excitatory synaptic plasticity (i.e., NMDAR-dependent LTD) of NAcSh D1MSNs in ethanol-induced neuroadaptations. Together, the results of the behavioral, genetic, pharmacological, and electrophysiological experiments described here support the notion that NAcSh LTD measured in D1MSNs *ex vivo* is a meaningful correlate of *in vivo* ethanol consumption, and further validate ALK as a promising target for the treatment of AUD.

## Materials and Methods

### Animals

This study was carried out in accordance with the recommendations of the “Guidelines for the Care and Use of Animals in Neuroscience” issued by the National Academies. The protocols were approved by the Institutional Animal Care and Use Committee of The University of Texas at Austin.

To generate *Alk*^KO^ mice for behavioral experiments, we started a colony by crossing male *Alk*^KO^ mice (Lasek et al., 2011b; a gift from Dr. Y. Blednov, UT Austin) with female C57BL/6J mice (purchased directly from The Jackson Laboratory, Stock No. 000664). This colony was then maintained by crosses of *Alk*^het^ × *Alk*^het^ or *Alk*^het^ × *Alk*^KO^. An existing colony of *Drd1a-*tdTomato mice (Ade et al., 2011; initial breeding pairs obtained from The Jackson Laboratory, Stock No. 016204) was maintained by crosses in which only one parent (typically the male) was a carrier of the *Drd1a-*tdTomato transgene. To generate *Alk*^KO^ carriers of the *Drd1a-*tdTomato transgene (*Drd1a-*tdTomato^hemi^/*Alk*^KO^) for electrophysiology experiments, female *Alk*^KO^ mice from the initial *Alk* colony were crossed with male *Drd1a-*tdTomato hemizygous (*Drd1a-*tdTomato^hemi^) mice from the main *Drd1a-*tdTomato colony. Offspring from these crosses were used in breeding pairs of *Drd1a-*tdTomato^hemi^/*Alk*^het^ males and *Drd1a-*tdTomato^negative^/*Alk*^het^ females, and this *Drd1a*-tdTomato/*Alk* line continued to be maintained using crosses in which only one parent (typically the male) was a carrier of the *Drd1a-*tdTomato transgene. Behavioral experiments used *Alk*^KO^ and *Alk*^WT^ mice from the *Alk* colony, and *Drd1a-*tdTomato^negative^ and *Drd1a-*tdTomato^hemi^ mice from the *Drd1a-*tdTomato colony. Electrophysiological experiments with *Drd1a-*tdTomato^hemi^/*Alk*^KO^ mice used *Drd1a*-tdTomato^hemi^/*Alk*^WT^ littermates for comparison. All other electrophysiological experiments used *Drd1a-*tdTomato^hemi^ mice from the *Drd1a-*tdTomato colony and the *Drd1a*-tdTomato/*Alk* line.

Male mice were used for all experiments. Mice used for behavior were approximately 9–16 weeks old when the ethanol pre-exposure phase began (see below), and mice were used for electrophysiology at approximately 7–17 weeks old. Except as described below in Section “Behavior,” experimental mice were group-housed (up to five mice per cage) in standard cages (7.5″ × 11.5″ × 5″) with Sani-Chips wood bedding (PJ Murphy) and a cotton fiber nestlet (Ancare), in a temperature controlled room (∼70°F) with lights on at 12:00 am and off at 12:00 pm, and had *ad libitum* access to standard chow (LabDiet^®^5LL2 Prolab RMH 1800) and a single bottle of tap water. With the exception of the ethanol pre-exposure sessions, which started 30 min prior to the onset of the dark cycle, all behavioral procedures and electrophysiological experiments took place during the dark cycle.

### Behavior

At least 3 weeks prior to beginning operant training, mice were transferred from group holding cages and housed as one pair per cage (generally one of each genotype). Mice were assigned aliases at the time of transfer from group to pair-housing to blind experimenters to the genotype. Paired housing cages were identical to group housing, with the exception that from this point on, water was available in the home cage from either of two 50 ml polystyrene tubes (Fisher) fitted with rubber stoppers (size 6) and 2.5″ long metal sippers (OT-100, Ancare) that inserted vertically through the cage top. Mice were allowed at least 1 week to acclimate to these new housing conditions.

Ethanol drinking solution (15%, v/v) was prepared by diluting 190 proof pure ethyl alcohol (Sigma-Aldrich) in tap water, and was stored in a light-protected glass bottle at room temperature. Mice were weighed a minimum of once per week. Behavioral procedures were conducted on any day of the week, but never for more than five consecutive days.

#### Ethanol Pre-exposure

Approximately 2 weeks prior to operant training, mice first were exposed to ethanol during daily two-bottle choice sessions (nine sessions total). Approximately 1 h before the onset of the dark cycle, mice were separated into individual cages, which had no nestlet or water bottles, but *ad libitum* access to food. At 30 min prior to the dark cycle, two bottles – one containing 15% ethanol and the other tap water (both identical in construction to those used for home cage water access) – were inserted into the cage top. After 2 h the bottles were removed and mice were returned to their home cages. The relative positions of the bottles (right vs. left) were alternated each session. Consumption of 15% ethanol and water was determined by weighing the bottles before and after each drinking session. Preference for 15% ethanol was calculated by dividing ethanol solution consumption by total solution (water + ethanol) consumption.

#### Operant Self-administration

##### Operant chambers and programming

Operant conditioning chambers (modular test chamber, ENV-307A) were housed inside sound-attenuating cubicles (ENV-022MD) from Med Associates, Inc. (Georgia, VT, United States). Chambers were equipped with Med Associates components: a house light (100 mA; ENV-315M) located at the top center of the left wall (illuminated during all sessions), two stimulus lights (ENV-321M) – one above each of two retractable levers (ENV-312-2M) located on the proximal and distal sections of the right wall, a retractable sipper tube assembly (ENV-352AW) positioned between the levers, and a metal grid floor (ENV-307A-GFW). Tubes to hold the ethanol drinking solution were made in the lab by cutting off the ends of 10 mL graduated, polystyrene serological pipettes, and inserting at one end a 2.5″ long, ball point metal sipper tube (TD-100, Ancare) that was secured with superglue and parafilm. Silicone stoppers (size 10D; Fisher) were used to seal the other end. Pipette graduations were used to read the volume of ethanol solution in the tube to the nearest 0.05 mL, which was manually recorded before and after each session.

Programs written for Med-PC IV software (Med Associates, Inc., Georgia, VT, United States) controlled all chamber components and recorded the occurrence and time of events of interest – active and inactive lever presses, insertions of the metal sipper tube into the chamber (each insertion was considered one “reinforcer” earned). The side of active lever (right vs. left) was equally distributed between the two genotypes. All operant programs were written so that whenever the sipper tube inserted into the chamber, the active lever retracted (and then reinserted upon sipper retraction). For all five conditioning sessions, the first two self-administration sessions, and the PR session, the stimulus light above the active lever flashed briefly (5 Hz for 1 s) when a reinforcer was earned.

##### Operant conditioning and testing

After the ethanol pre-exposure phase, mice were habituated to drinking ethanol in the operant chambers over four sessions (one per day, each 2 h long), during which the levers were retracted, and the metal sipper tube containing 15% ethanol was inserted into the chamber for the entire session. Approximately 20 h before the next session in the operant chamber, mice were separated into individual cages. Over the next five sessions (one session per day, each lasting 2–3 h), mice were conditioned to press one of the two levers (“the active lever”) using a fixed ratio 1 (FR1) reinforcement schedule (one press of the active lever granted one “reinforcer” – insertion of the sipper tube for 20 s). In addition, the first two of these sessions also granted intermittent, non-contingent insertions of the sipper tube. For the first two conditioning sessions, mice were water restricted for approximately 18–20 h beforehand; for the next three, they were restricted for approximately 4–6 h beforehand. After the last of these five conditioning sessions, mice were no longer water restricted and were returned to pair housing. All subsequent operant self-administration sessions lasted 1 h, and variations in the progression of reinforcement schedules that were in effect for each 1 h operant self-administration session are described below.

In the experiment comparing *Alk*^WT^ with *Alk*^KO^ mice (reported in Effects of *Alk* Deletion on Operant Ethanol Self-Administration), the reinforcement schedule increased from FR1 (one session) to FR2 (three sessions), then FR3 (two sessions), then FR4, after which mice then received nine additional sessions of FR4 (10 FR4 sessions total). Any mouse that did not drink at least 0.5 g ethanol per kg body weight on at least two of the final five FR4 sessions was excluded from statistical analyses (*n* = 3 *Alk*^KO^, 2 *Alk*^WT^). One week after the last FR4 session, mice were tested in a 1-h self-administration session under a progressive ratio (PR) schedule of reinforcement: the required number of active lever presses to receive one reinforcer started with four, but then increased by two presses each time reinforcement was earned.

In the experiment to evaluate *ex vivo* LTD following operant ethanol self-administration experience (reported in Disruption of LTD in NAc D1MSNs Following Operant Self-Administration of Ethanol), the reinforcement schedule in effect for each session also progressed from FR1 to FR2, FR3, then FR4. However, the number of sessions under each schedule was not fixed, but was determined by behavioral performance: the number of active lever presses had to exceed 50% of the total lever presses for two consecutive sessions before advancement to the next reinforcement schedule. Mice were then maintained on the FR4 reinforcement schedule for 6–18 sessions, with the exception of one mouse that never advanced beyond FR2. The total number of operant sessions ranged from 21 to 34. Twenty-four hours after the final self-administration session, brain slices containing the NAc were prepared for electrophysiology.

### Electrophysiology

#### Brain Slice Preparation and Recording Conditions

Mice were lightly anesthetized by isoflurane inhalation, then decapitated, and brains were rapidly removed and placed in ice-cold (4°C) oxygenated high-sucrose artificial cerebrospinal fluid (ACSF) containing the following (in mM): 210 sucrose, 26.2 NaHCO_3_, 1 NaH_2_PO_4_, 2.5 KCl, 6 MgCl_2_, 2.5 CaCl_2_, 11 dextrose, bubbled with 95% O_2_/5% CO_2_. Parasagittal slices (235–250 μm thick) containing the nucleus accumbens were sectioned in ice-cold, high-sucrose ACSF using a Leica VT1000S vibrating microtome. Slices were transferred to a light-protected recovery bath of ACSF containing in mM: 124 NaCl, 26 NaHCO_3_, 1 NaH_2_PO_4_, 4.4 KCl, 2.4 MgCl_2_, 1.8 CaCl_2_, 10 dextrose, bubbled with 95% O_2_/5% CO_2_, at 32°C, and maintained for at least 1 h before transferring to the recording chamber.

All recordings were conducted at 31–33°C in ACSF containing (in mM) 124 NaCl, 26 NaHCO_3_, 1 NaH_2_PO_4_, 4.4 KCl, 1.2 MgCl_2_, 2 CaCl_2_, 10 dextrose, and 0.05 picrotoxin, bubbled with 95% O_2_/5% CO_2_, and continuously pumped into the recording chamber at ∼2 mL/min. For experiments with the ALK inhibitor TAE684 (ActiveBiochem), frozen aliquots of TAE684 (in 100% DMSO) were defrosted and added to the recording ACSF to make final concentrations of 5 or 25 nM (final concentration of DMSO in both cases: 0.00125%). Slices were transferred to the recording chamber and incubated in TAE684- or 0.00125% DMSO (vehicle)-containing ACSF for at least 30 min before beginning experiments. Recording electrodes (resistances ∼3–7 MΩ) were made from 4″ thin-wall glass (1.5 OD/1.12 ID; World Precision Instruments) using a P-97 Flaming/Brown micropipette puller (Sutter Instruments) and contained (in mM): 135 KMeSO_4_, 12 NaCl, 0.5 EGTA, 10 HEPES, 2 Mg-ATP, 0.3 Tris-GTP, ∼280–295 mOsm (pH 7.3 with KOH). Unless otherwise noted, chemicals were obtained from Sigma-Aldrich or Fisher Scientific.

#### Data Acquisition

Whole-cell patch clamp recordings were acquired with a CV203BU headstage and Axopatch 200B amplifier, filtered at 1 kHz, and digitized at 5 kHz *via* a Digidata 1440A interface board using Clampex 10.3 (all products by Molecular Devices, Sunnyvale, CA, United States). Neurons in the ventromedial NAcSh were identified as D1MSNs by epifluorescent illumination of tdTomato using the MRK200 Modular Imaging system (Siskiyou Corporation, Grants Pass, OR, United States) mounted on a vibration isolation table and selected for experimentation based on parameters measured just after obtaining whole-cell configuration: series resistance < 30 MΩ, resting membrane potential ≤-70 mV, and membrane voltage responses to current injections consistent with features previously reported for MSNs (inward rectification at hyperpolarized potentials and slow-ramp depolarization preceding first action potential fired in response to positive current injection). Series resistance was monitored throughout experiments, and the recording was terminated if this resistance varied by more than 20%, or exceeded 30 MΩ. Changes in the holding current were monitored to detect instability of the patch.

Membrane voltage responses to hyperpolarizing and depolarizing current injections were measured by applying 300 ms intracellular pulses in 50 pA steps from -400 to 400 pA, at 700 ms start-to-start. AMPAR-mediated EPSCs (evoked or spontaneous) were monitored by clamping the membrane potential at -80 mV. For plasticity experiments, excitatory afferents were locally stimulated with a stainless steel bipolar stimulating electrode (MX21AES, FHC, Inc., Bowdoin, ME, United States) placed ∼100 μm anterodorsal to the cell body. Evoked EPSCs were recorded for at least 10 min (at 0.1 Hz) to ensure stable baseline amplitudes before delivering the LTD induction (“pairing”) protocol for synaptic conditioning: 500 stimulations at 1 Hz paired with continuous post-synaptic membrane depolarization to -50 mV. Evoked EPSCs were monitored at 0.1 Hz for 30 min post-pairing. Only one cell per slice was used for plasticity experiments. Raw data analysis was done in Clampfit 10.3 (Molecular Devices). Frequency and average amplitude of spontaneous EPSCs were determined (using Clampfit Template Search) for a 3-min period beginning a few minutes after achieving whole-cell configuration, prior to evoking EPSCs. Events with amplitudes < 5 pA were rejected and cells in which event frequency was < 0.5 Hz were excluded from statistical analyses.

### Statistical Analyses

IBM SPSS Statistics 23 was used to perform General Linear Model Repeated Measures, One-Way ANOVA, Independent Samples *t*-test, or Bivariate Correlations procedures as appropriate. Welch’s *t*-test or adjusted *F*-statistic were used, as indicated in the text, when homogeneity of variance between groups was violated. The Greenhouse–Geisser adjusted degrees of freedom were used, and are given in the text, when sphericity within groups was violated. For plasticity analyses, the amplitude of each evoked EPSC was normalized as a percentage of the baseline average for that individual recording. NMDAR-dependent LTD of AMPAR-mediated EPSCs in D1MSNs in the NAcSh is expressed during the 10 min period lasting 20–30 min post-pairing (Jeanes et al., 2014). Analyses of within-group effects (main or simple effects of pairing) compared this 10-min LTD period to the 10-min baseline using 2-min bin averages. Significant within (pairing) × between (genotype or concentration) interaction effects were further investigated by analyzing the simple effect of pairing for each condition (genotype or concentration), and the simple effect of condition (genotype or concentration) during the LTD (hereafter referred to as “post-pairing”) phase. For simple effects analyses, *F*-value and significance were computed using the MS_error_ and df from the overall analysis (Kirk, 1982). Statistical significance was set at *p* < 0.05. Unless stated otherwise, *post hoc* comparisons used the Bonferroni correction to maintain *p* < 0.05 Type I error rate for each group of tests.

## Results

### Effects of Alk Deletion on Operant Ethanol Self-administration

As *Alk*^KO^ mice have only been tested for ethanol consumption in home cage access paradigms, we sought to further characterize their drinking phenotype using a different model – operant self-administration. Prior to beginning operant training, we pre-exposed mice to ethanol during 2-h sessions in which they could drink either water or 15% ethanol. Preference for ethanol during these sessions was similar between the two genotypes and increased for both groups over time [*F*_GENOTY PE_(1,28) = 0.03, n.s.; *F*_SESSION_(8,224) = 13.22, *p* < 0.001; *F*_GENOTY PE_ × _SESSION_ (8,224) = 0.80, n.s. data not shown]. Average total fluid intake per session (*Alk*^WT^: 20.1 ± 1.2 g/kg, *Alk*^KO^: 22.5 ± 1.6 g/kg) and ethanol dose consumed per session (*Alk*^WT^: 1.7 ± 0.2 g/kg, *Alk*^KO^: 1.9 g/kg ± 0.2 g/kg) also did not differ between genotypes [*t*(28) = 1.17, n.s., total fluid, 0.77, n.s., ethanol dose].

**FIGURE 1:**
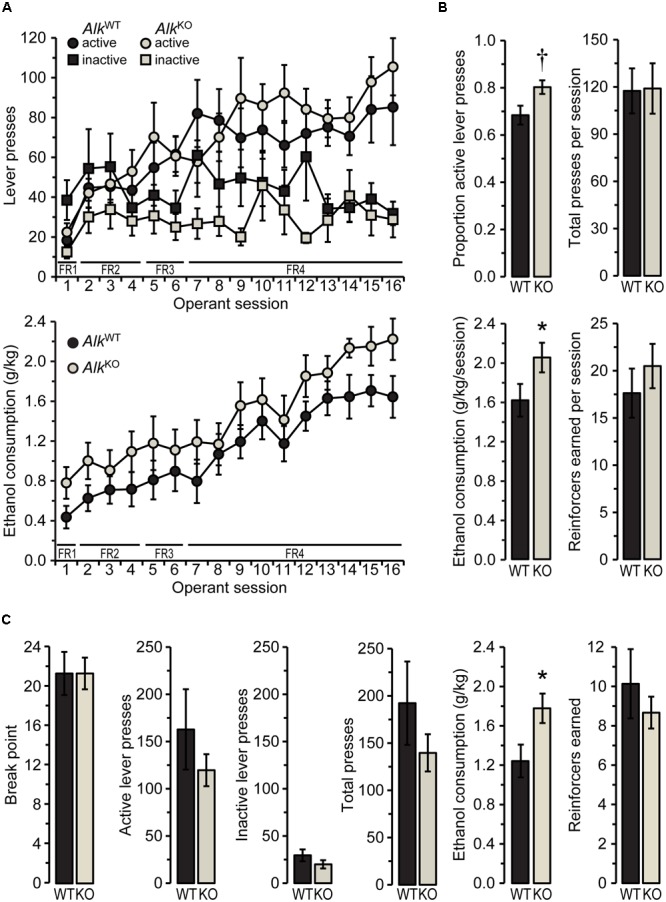
*Alk*^KO^ mice drink more ethanol than *Alk*^WT^ during operant self-administration sessions. **(A)** Active and inactive lever presses (top) and ethanol consumption (bottom) during all 1-h operant sessions with fixed ratio (FR) schedules of reinforcement (required ratio for each session is indicated along x-axis). **(B)** Values shown are averages of the final five FR4 sessions. ^†^*p* = 0.07, ^∗^*p* = 0.047, *Alk*^KO^ vs. *Alk*^WT^. **(C)** Lever pressing and reinforcement during the progressive ratio (PR) test. ^∗^*p* = 0.02, *Alk*^KO^ vs. *Alk*^WT^. All data shown as group means ± SEM; *n* = 15 for each genotype.

Mice then were conditioned in operant self-administration chambers to press one of two levers (the “active” lever) for the opportunity to drink from a sipper containing 15% ethanol. Both genotypes showed increasing preference for the active lever over time, as the lever press requirement increased from FR1 to FR4 [*F*_SESSION_(15,390) = 5.17, *p* < 0.00001, *F*_GENOTY PE_ × _SESSION_(15,390) = 0.97, n.s.], and although *Alk*^KO^ mice tended to exhibit a greater preference for the active lever, relative to *Alk*^WT^, the difference was not statistically significant [*F*_GENOTY PE_(1,26) = 3.39, *p* = 0.08, **Table 1**]. Indeed, the average number of active, inactive, and total lever presses per session did not differ between the two genotypes over all 1-h FR self-administration sessions, nor did the number of reinforcers earned per session (**Figure 1A** and **Table 1**). This pattern of results indicates that the level of general activity was similar and the ability of ethanol to serve as a reinforcer of lever pressing behavior was comparable between the two genotypes. Nevertheless, when the dose of ethanol consumed per session reached stable levels during the final five FR4 sessions [*F*_SESSION_(4,112) = 2.17, n.s.], *Alk*^KO^ mice drank more ethanol per session overall [*F*_GENOTY PE_(1,28) = 4.32, *p* = 0.047; **Figures 1A,B**]. Notably, this difference in ethanol consumption was not accompanied by differences in lever pressing or reinforcers earned [*F*_GENOTY PE_(1,28) = 3.59, *p* = 0.07, active lever preference, 0.01, n.s., total lever presses, and 0.67, n.s., reinforcers earned], **Figure 1B**.

**Table 1:** Operant self-administration measures in *Alk*^WT^ vs. *Alk*^KO^ mice during fixed ratio sessions.

Measure	*Alk*^WT^	*Alk*^KO^	*F*	*p*
	Mean ± SEM	Mean ± SEM		
Active lever presses	64 ± 10	71 ± 9	*F*(1,28) = 0.25	0.62
Inactive lever presses	44 ± 9	29 ± 6	*F*(1,28) = 1.86	0.18
Total lever presses	108 ± 14	100 ± 14	*F*(1,28) = 0.17	0.68
Active lever preference	0.62 ± 0.04	0.73 ± 0.04	*F*(1,26) = 3.39^1^	0.08
Reinforcers earned	17.2 ± 2.7	19.0 ± 2.7	*F*(1,28) = 0.23	0.64

*Data are per session values, averaged over all 16 1-h fixed ratio operant self-administration sessions. ^1^WS df = 26 due to two subjects being excluded from this analysis for having no lever presses during one session.*

After the last of these FR sessions, mice received 1 week of forced abstinence before testing for operant self-administration under a PR schedule of reinforcement. PR schedules reveal differences in the motivational properties of a reinforcer, such as ethanol, by requiring an increasing number of lever presses after each reinforcer is earned (Ripley and Stephens, 2011). The results of the PR test (shown in **Figure 1C**) indicated similar motivation to obtain ethanol for the two genotypes, as there was no difference in the break point [the final ratio requirement, i.e., the number of active lever presses required for one reinforcer, *t*(28) = 0.00, n.s.]. Active, inactive, and total lever presses also did not differ between genotypes [*t*(28) = 0.94, 1.2, and 1.1, respectively, all n.s.]. Similarly to the previous FR4 operant sessions, however, *Alk*^KO^ mice drank significantly more ethanol during the PR session [*t*(28) = 2.39, *p* = 0.024], despite earning a similar number of reinforcers [*t*(28) = 0.76, n.s.].

### Electrophysiological Effects of Alk Deletion or ALK Inhibition on NAcSh D1MSNs

#### Membrane Properties and Spontaneous Excitatory Transmission

To our knowledge, no other studies have evaluated how ALK influences cellular physiology or synaptic transmission in the nucleus accumbens. Thus, we began our electrophysiological studies by determining the effect that loss of ALK has on cellular membrane properties and excitability, and on spontaneous excitatory synaptic transmission in the NAcSh. MSNs expressing dopamine D1 versus dopamine D2 receptors exhibit differences in a number of electrophysiological properties and prior work from our lab showed that these MSN subtypes are differentially affected by chronic ethanol exposure (Planert et al., 2013; Jeanes et al., 2014; Renteria et al., 2016b). Therefore, we crossed our *Alk* line with the *Drd1a-*tdTomato line of mice (Ade et al., 2011) so that we could record specifically from tdTomato-positive (presumed dopamine D1 receptor-expressing) MSNs of *Alk*^KO^ and *Alk*^WT^ mice (refer to Animals for details).

**FIGURE 2:**
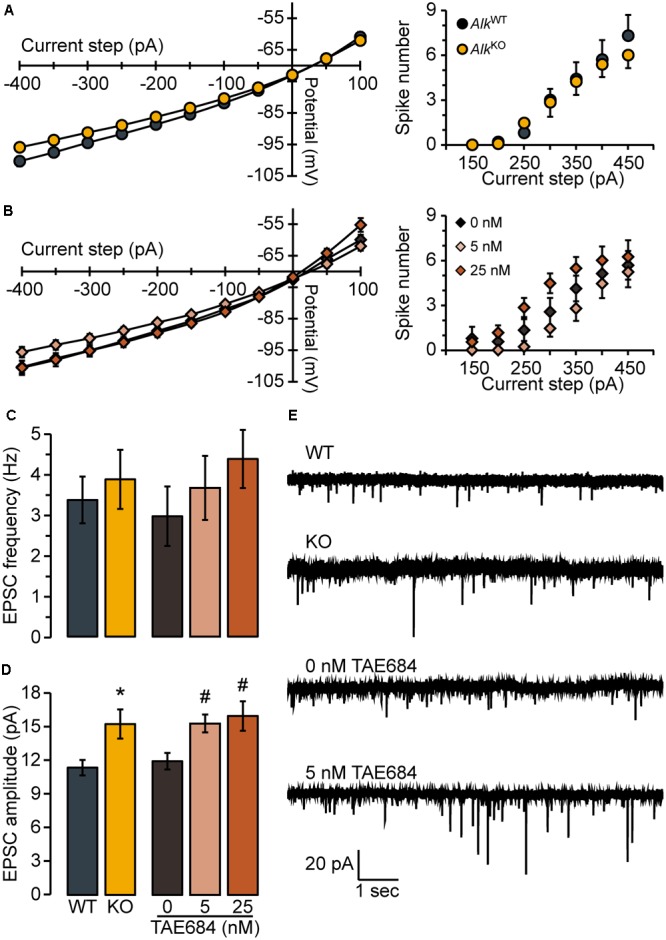
Loss of ALK does not alter membrane properties or excitability but promotes excitatory synaptic transmission. Membrane voltage (left) and spike firing (right) responses to hyperpolarizing and depolarizing current steps (300 ms duration) for *Alk*^KO^ vs. *Alk*^WT^ **(A)** or TAE684 (5 and 25 nM) vs. vehicle (0 nM) **(B)**. Frequency **(C)** and amplitude **(D)** of spontaneous EPSCs in *Alk*^WT^ and *Alk*^KO^, and in vehicle-treated (0 nM) and TAE684-treated (5 and 25 nM). ^∗^*p* = 0.02, *Alk*^KO^ vs. *Alk*^WT^. ^#^*p* = 0.02, 5 nM vs. 0 nM, and *p* = 0.04, 25 nM vs. 0 nM (Games-Howell *post hoc* comparisons). **(A–D)** Data are represented as group means ( ± SEM) for *Alk*^WT^ (*n* = 10 cells, four mice), *Alk*^KO^ (*n* = 13 cells, five mice), vehicle-treated (*n* = 8 cells, five mice), 5 nM TAE684 (*n* = 9 cells, five mice), and 25 nM TAE684 (*n* = 14 cells, six mice). **(E)** Representative current traces showing NAcSh D1MSN spontaneous EPSCs.

We found that membrane voltage responses and action potential spike firing to hyperpolarizing and depolarizing current steps appeared normal in D1MSNs from *Drd1a-*tdTomato^hemi^/*Alk*^KO^ mice, compared to *Drd1a-*tdTomato^hemi^/*Alk*^WT^ [membrane voltage responses: *F*_GENOTY PE_(1,21) = 3.6, n.s., *F*_GENOTY PE_ × _STEP_(1.1,23.5) = 2.1, n.s.; spike firing: *F*_GENOTY PE_(1,21) = 0.071, n.s., F_GENOTY PE_ × _STEP_(2.1,44.4) = 0.41, n.s.; **Figure 2A**]. We next examined whether acute pharmacological inhibition of ALK also would not affect these properties by pretreating brain slices from *Drd1a-*tdTomato^hemi^ mice with the selective ALK inhibitor, TAE684 (Galkin et al., 2007), or its vehicle, DMSO (0.00125%). Membrane voltage responses were not affected by TAE684 pretreatment [*F*_CONCENTRATION_(2,27) = 1.3, n.s., *F*_CONCENTRATION_ × _STEP_(2.2,30.1) = 2.4, n.s.; **Figure 2B**, left]. Analysis of spike firing did reveal a statistically significant main effect of concentration [*F*(2,27) = 3.8, *p* = 0.034, **Figure 2B**, right], but *post hoc* analyses found neither concentration of TAE684 tested to be different from vehicle. As shown in **Figures 2C,E**, the frequency of AMPA receptor-mediated spontaneous EPSCs was not different in *Drd1a-*tdTomato^hemi^/*Alk*^KO^ mice [*t*(20) = 0.51, n.s.], and was unaffected by TAE684 pretreatment [*F*(2,28) = 0.86, n.s.]. In contrast, the average amplitude of spontaneous EPSC events was greater in *Drd1a-*tdTomato^hemi^/*Alk*^KO^ mice, relative to *Drd1a-*tdTomato^hemi^/*Alk*^WT^ [Welch’s *t*(21) = 2.7, *p* = 0.016], and was affected similarly by TAE684-pretreatment [Welch’s *F*(2,18.6) = 6.1, *p* = 0.0091], **Figures 2D,E**. As changes in event frequency are generally believed to reflect presynaptic effects of a manipulation, and changes in amplitude to reflect post-synaptic effects, these results – although certainly not conclusive – are suggestive of a post-synaptic site of action.

#### LTD of AMPAR-Mediated Excitatory Synaptic Transmission

**FIGURE 3:**
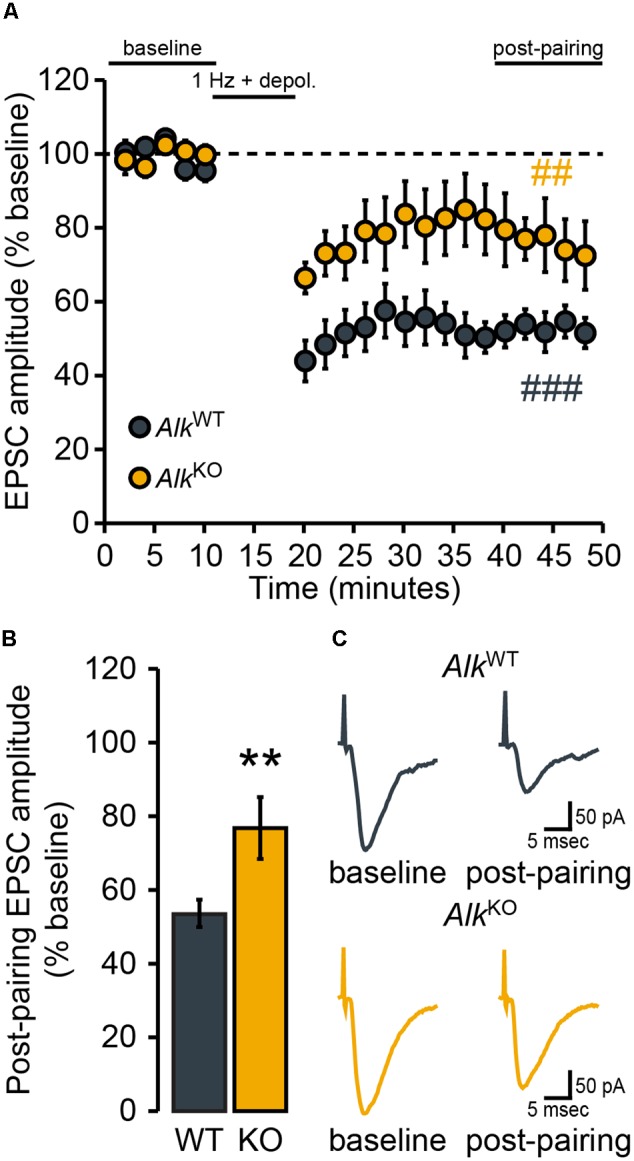
NAcSh D1MSN LTD is attenuated in *Alk*^KO^ mice. D1MSNs from *Alk*^WT^ mice (*n* = 5 cells, four mice) showed robust LTD of evoked EPSCs over the “post-pairing” period (40–50 min). In comparison, the magnitude of LTD expressed was markedly less in D1MSNs from *Alk*^KO^ mice (*n* = 6 cells, three mice). **(A)** Data points are group means ( ± SEM) for normalized (% baseline) EPSC amplitudes averaged over 2-min bins. ^###^*p* = 0.0001, post-pairing vs. baseline period (simple effect of pairing) in *Alk*^WT^. ^##^*p* = 0.007, post-pairing vs. baseline period (simple effect of pairing) in *Alk*^KO^. **(B)** Bars show group means ( ± SEM) for normalized EPSC amplitudes averaged over the entire 10-min post-pairing period. ^∗∗^*p* = 0.004, *Alk*^KO^ vs. *Alk*^WT^ (simple effect of genotype during post-pairing period). **(C)** Representative traces of evoked EPSCs during the baseline and post-pairing periods.

Given that the expression of NMDAR-dependent LTD of evoked AMPAR EPSCs in NAc MSNs (hereafter just referred to as “LTD”) has been widely implicated in responses to drugs of abuse, including ethanol (see Introduction), we next determined whether loss of ALK (*Alk* deletion or ALK inhibition) affected the expression of this form of plasticity. Following the delivery of synaptic conditioning stimuli (the “pairing” protocol described in Data Acquisition), NAcSh D1MSNs from *Drd1a-*tdTomato^hemi^/*Alk*^WT^ mice showed the expected robust depression EPSC amplitude during the period lasting from 20 to 30 min post-pairing (**Figures 3A,C**). For NAcSh D1MSNs from *Drd1a-*tdTomato^hemi^/*Alk*^KO^ mice, LTD expression was attenuated, although not completely absent (**Figures 3A,C**). Mixed model, repeated measures analysis of genotype and pairing effects indicated that significant depression of evoked EPSC amplitudes occurred for both genotypes, but the effect of pairing was greater for *Drd1a-*tdTomato^hemi^/*Alk*^WT^ mice [*F*_PAIRING_(1,9) = 50.2, *p* < 0.0001; *F*_GENOTY PE_ × _PAIRING_ (1,9) = 5.6, *p* = 0.04]. Furthermore, the magnitude of LTD was significantly different between the two genotypes (**Figure 3B**). In other words, constitutive, global loss of *Alk* was associated with reduced LTD magnitude in NAcSh D1MSNs.

**FIGURE 4:**
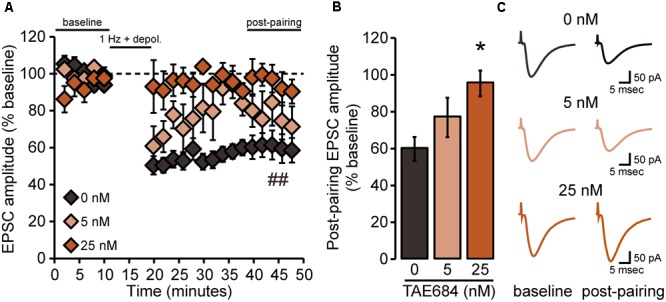
NAcSh D1MSN LTD is attenuated by pharmacological inhibition of ALK. **(A)** Data points are group means ( ± SEM) for normalized (% baseline) EPSC amplitudes averaged in 2-min bins; vehicle (0 nM: *n* = 6 cells, six mice; 5 nM TAE684: *n* = 4 cells, three mice; 25 nM TAE684: *n* = 4 cells, three mice). In the vehicle group alone, evoked EPSC amplitudes during the 40–50 min “post-pairing” period were significantly reduced relative to baseline. ^##^*p* = 0.0003, post-pairing vs. baseline period (simple effect of pairing in vehicle group). **(B)** Bars show group means ( ± SEM) for normalized EPSC amplitudes averaged over the entire 10-min post-pairing period. 25 nM TAE684 was significantly different from 0 nM (^∗^*p* = 0.02), while 5 nM TAE684 was not different from either concentration (Bonferroni *post hoc* comparisons). **(C)** Representative traces of evoked EPSCs during the baseline and post-pairing periods.

We then determined whether pharmacological inhibition of ALK also would alter the expression of LTD in NAcSh D1MSNs by pretreating brain slices from *Drd1a-*tdTomato^hemi^ mice with the selective ALK inhibitor TAE684 (**Figure 4**). Statistical analysis indicated an interaction [*F*_CONCENTRATION_ × _PAIRING_(2,11) = 5.9, *p* = 0.02] of the two main effects, pairing [*F*(1,11) = 16.8, *p* = 0.002] and TAE684 concentration [*F*(2,11) = 4.7, *p* = 0.03] (**Figure 4A**). Further investigation of this interaction revealed a statistically significant effect of pairing only for the vehicle group (**Figure 4A**), and that the effect of TAE684 on the magnitude of LTD was concentration-dependent [*F*_CONCENTRATION_(2,11) = 5.3, *p* = 0.03; **Figure 4B**]. In summary, the brain slice electrophysiology experiments found that loss of ALK activity did not influence passive membrane properties or membrane excitability of NAcSh D1MSNs, but did enhance excitatory transmission and alter the expression of excitatory synaptic plasticity in these neurons.

### Disruption of LTD in NAc D1MSNs Following Operant Self-administration of Ethanol

In the experiments described in the preceding sections, we observed relationships between *Alk* deletion and greater consumption of ethanol under operant self-administration conditions, and between ALK and the magnitude of LTD induced *ex vivo* in NAcSh D1MSNs – results that are consistent with our working model that the observation of altered LTD expression may be a predictor of greater ethanol consumption. Conversely, we have reported elsewhere that *in vivo* ethanol exposure in the CIE vapor model – which produces an escalation of non-operant self-administration of ethanol (Griffin et al., 2009) – also produces a loss of the LTD that normally can be induced in NAcSh D1MSNs *ex vivo* (Jeanes et al., 2014; Renteria et al., 2016b, 2017). Taken all together, these findings are suggestive of a bidirectional relationship between the mechanisms that underlie *ex vivo* LTD and *in vivo* ethanol exposure, but many questions remain. For example, do all forms of ethanol experience (e.g., active vs. passive, operant vs. non-operant) affect glutamatergic synaptic plasticity similarly, or, put another way, is ethanol exposure-induced glutamatergic metaplasticity dependent on dose or route of administration? Of specific relevance to the present work is the matter of whether operant ethanol self-administration is sufficient to disrupt LTD in NAcSh D1MSNs *ex vivo*.

**FIGURE 5:**
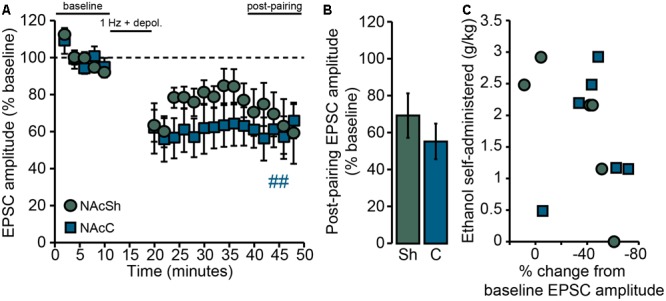
Loss of LTD in NAcSh D1MSNs following operant self-administration of ethanol is correlated with ethanol consumption. **(A)** Data points are group means ( ± SEM) for normalized (% baseline) EPSC amplitudes averaged in 2-min bins; NAcSh: *n* = 6 cells, five mice; NAcC: *n* = 6 cells, six mice. Half of all cells were pairs of recordings made in two different slices (one core, one shell) from the same mouse. ^##^*p* = 0.005 for NAcC post-pairing vs. baseline period. **(B)** Bars show group means ( ± SEM) for normalized EPSC amplitudes averaged over the entire 10-min post-pairing period. **(C)** Data points are individual observations for the cells that comprise the group data shown in **(A,B)**. For NAcSh D1MSNs (circles) there was a strong, inverse correlation between ethanol consumption during the final operant self-administration session and the magnitude of LTD expressed 24 h later (*r*_s_ = –0.93, *p* = 0.008). NAcC D1MSNs (squares) did not show such a relationship (*r*_s_ = 0.09, n.s.).

To address this question, *Drd1a-*tdTomato^hemi^ mice were trained for operant self-administration of ethanol (described in Behavior) and LTD was evaluated *ex vivo* 24 h after the final self-administration session. Following delivery of the synaptic conditioning protocol (the “pairing” protocol described in Data Acquisition), the amplitudes of post-pairing EPSCs in NAcSh D1MSNs tended to be reduced from baseline, but the effect of pairing (i.e., the depression of evoked AMPAR EPSCs) was not at the level of statistical significance [*F*_PAIRING_(1,5) = 4.05, *p* = 0.1; **Figure 5A**]. Further, the mean post-pairing EPSC amplitude (∼70% of baseline; **Figure 5B**) was apparently greater than what we usually observe for NAcSh D1MSNs from ethanol-naïve *Drd1a-*tdTomato mice (∼50–60% of baseline, see Figures 3, 4, as well as Figure 1 of Renteria et al., 2016b). In interpreting this result, we reasoned that if the loss of LTD is an ethanol dose-dependent phenomenon, then the magnitude of LTD observed *ex vivo* should be inversely related to *in vivo* ethanol consumption. In other words, given that self-administered doses ranged from essentially 0 g/kg to nearly 3 g/kg, the magnitude of LTD should vary accordingly. Indeed, as shown in **Figure 5C**, we found that there was strong, negative correlation between the ethanol dose consumed during the final operant self-administration session and the magnitude of LTD observed in NAcSh D1MSNs [*r*_s_(4) = -0.93, *p* = 0.008]. This does not appear to be a non-specific relationship between operant ethanol consumption and LTD in general, however, as D1MSNs in the core of the NAc (NAcC) still exhibited LTD [*F*_PAIRING_(1,5) = 22.1, *p* = 0.005; **Figures 5A,B**], and LTD magnitude was not correlated to ethanol consumption [*r*_s_(4) = 0.09, n.s.; **Figure 5C**]. We do note, though, that this lack of correlation should be interpreted with caution, given the small sample size.

## Discussion

Here, we have combined behavioral and electrophysiological approaches to study ALK as a potential target for alcohol medication development. ALK, a receptor tyrosine kinase, has previously been implicated in a number of psychiatric conditions – schizophrenia, depression, anxiety, and AUDs/SUDs (Kunugi et al., 2006; Bilsland et al., 2008; Lasek et al., 2011b; Wang et al., 2011; Weiss et al., 2012; He et al., 2015; Schweitzer et al., 2016; Dutton et al., 2017). We extended the characterization of the behavioral phenotypes of *Alk*^KO^ mice to an operant self-administration paradigm, allowing us to examine both ethanol drinking and seeking. The present finding of elevated ethanol consumption by *Alk*^KO^ mice during operant self-administration sessions is consistent with prior reports showing greater ethanol consumption by these mice in other drinking models that measured intake during home cage, free-access drinking sessions (Lasek et al., 2011b; Schweitzer et al., 2016). Interestingly, however, we found that *Alk*^KO^ mice are similar to *Alk*^WT^ mice in respect to appetitive operant behavior. They showed comparable lever pressing under both fixed and PR testing, thereby earning the same number of opportunities to drink ethanol from the sipper tube. Despite this similarity, *Alk*^KO^ mice drank more ethanol when the sipper tube was available. This suggests that the elevated consumption of ethanol by *Alk*^KO^ mice results from a difference in the primary reinforcing properties of ethanol, but not a difference in the motivational properties of ethanol.

Until recently, little was known regarding how ALK activity, or lack thereof, influences neurophysiology and synaptic transmission. Our electrophysiological experiments suggest that ALK does not directly influence membrane excitability of NAcSh D1MSNs. Rather, ALK deletion and inhibition resulted in greater average amplitude spontaneous EPSCs relative to respective controls. This finding is suggestive of a role for ALK in the post-synaptic regulation of excitatory synaptic transmission, but alone is not conclusive. The location of ALK in mammalian synapses has not yet been determined, at least to our knowledge, but ALK was shown to be located post-synaptically in *Drosophila* larvae neuromuscular junctions (i.e., glutamatergic synapses) by Rohrbough and Broadie (2010). Further, Rohrbough et al. (2013) reported that loss of ALK function increased, while activation of ALK signaling decreased, transmission amplitudes in these synapses – findings consistent with our observation.

In addition to enhanced spontaneous excitatory transmission, genetic deletion and pharmacological inhibition of ALK produced glutamatergic synaptic metaplasticity in NAcSh D1MSNs. Given that expression of NMDAR-dependent LTD in accumbal MSNs requires the regulated endocytosis of post-synaptic GluA2 subunit-containing AMPARs (Brebner et al., 2005; Jeanes et al., 2014), a parsimonious interpretation of the latter finding is that ALK participates in the regulated endocytosis of these receptors. The convergence of several lines of evidence suggests an involvement of protein kinase C (PKC) in such a phenomenon. PKC activation promotes GluA2 internalization *via* phosphorylation of the GluA2 serine 880 residue and phosphorylation of this residue is necessary for the induction of NMDAR-dependent LTD in hippocampal neurons (Chung et al., 2000; Seidenman et al., 2003). Several groups have linked ALK signaling with activation of the phospholipase C-PKC pathway (Bai et al., 1998; Crockett et al., 2004; Wilson et al., 2015). Moreover, ALK inhibitors can block PKC activation and impair PKC-mediated phenomena, such as dopamine-induced dopamine D2 receptor (D2R) desensitization and D2R endocytosis (Donghong He and Amy Lasek, unpublished results; Dutton et al., 2017). Thus, we hypothesize that loss of ALK activity resulted in reduced activation of PKC during the LTD induction protocol, which impaired the regulated endocytosis of GluA2-containing AMPARs, thereby accounting for our observations of attenuated or absent LTD.

This is not to imply that ALK non-specifically promotes receptor endocytosis, however. Schweitzer et al. (2016) found no difference in the amplitude of GABAergic IPSCs recorded from neurons in the medial subdivision of the CeA (CeM) of *Alk*^KO^ and *Alk*^WT^ mice. They did report, though, that the frequency of these events was elevated in *Alk*^KO^ mice – an observation consistent with an effect of *Alk* deletion on presynaptic mechanisms. Considering that much of the GABAergic input onto CeM neurons is from centrolateral amygdala neurons that receive glutamatergic excitation by basolateral amygdala projection neurons (Gilpin et al., 2014), one plausible interpretation is that loss of *Alk* promotes glutamatergic signaling in this amygdalar circuit as well. In other words, one normal function of ALK may be to regulate the output of GABAergic neurons in multiple brain regions of the mouse (e.g., NAcSh, CeM) by attenuating glutamatergic excitation of these neurons. On the other hand, it is certainly conceivable that the effects of *Alk* deletion to increase presynaptic GABA release in the CeM and to enhance post-synaptic glutamate responses in the NAcSh could be mediated by entirely distinct mechanisms. Future studies will investigate these possibilities.

Another area for further research will be to determine the exact nature of the relationship between ALK activity, glutamatergic transmission in the NAcSh, and ethanol self-administration. Indeed, a limitation of the present work is that it does not allow us to make claims as to whether the effects of *Alk* deletion on glutamatergic transmission in NAcSh D1MSNs underlie the enhanced operant ethanol drinking. More generally speaking, we do not yet have a complete picture of how ALK activity in the NAcSh influences ethanol-drinking behaviors under different conditions. In fact, the ethanol-drinking phenotype of *Alk*^KO^ mice may be a consequence of developmental compensation (see Schweitzer et al., 2016 for discussion). As already mentioned, three studies have shown higher ethanol consumption by *Alk*^KO^ mice (in operant and non-operant paradigms). Yet systemic *in vivo* pre-treatment with ALK inhibitors actually reduced ethanol consumption in a non-operant model of self-administration, home cage “drinking-in-the-dark” (DID) (Dutton et al., 2017). When this finding is considered alongside our electrophysiological observations, a contradiction seems to arise: ALK deletion and inhibition appear to have the same effects on glutamatergic transmission in NAcSh D1MSNs, but may exert opposite effects on drinking behaviors. However, local knockdown of *Alk* in the NAcSh using RNA interference did not affect DID ethanol consumption, which implies that ALK activity in the NAcSh does not regulate drinking in that model. Thus, it may be the case that ALK in the NAcSh plays a role in ethanol consumption under operant, but not under non-operant, conditions. Finally, at present we cannot exclude the possibility that the effects of *Alk* deletion and ALK inhibition on glutamatergic transmission are only apparently similar manifestations of different downstream consequences of these manipulations.

In summary, the findings reported here contribute to a growing body of work implicating ALK as a newly discovered regulator of ethanol consumption. We found that *Alk* deletion appeared to alter the primary reinforcing properties of ethanol without affecting its motivational properties. Genetic deletion and pharmacological inhibition of ALK promoted excitatory synaptic transmission and impaired LTD of glutamatergic synapses on NAcSh D1MSNs. We and others previously have linked glutamatergic synaptic metaplasticity in the NAc to ethanol consumption and sensitization to the locomotor-stimulating effects of ethanol and psychostimulants (Brebner et al., 2005; Abrahao et al., 2013; Jeanes et al., 2014; Renteria et al., 2016a, 2017). These prior studies did not use operant self-administration paradigms, however, and focused on known components of glutamatergic transmission and signaling. Thus, the present work expands this field of inquiry in two ways. First, it demonstrates that operant self-administration of ethanol causes a loss in NAcSh LTD that is correlated to the amount of ethanol self-administered. Second, it identifies ALK as a possible, previously unknown molecular mediator of the relationship between glutamatergic synaptic metaplasticity in the NAc and alcohol-related behaviors, and supports the further investigation of ALK signaling as a potential novel therapeutic target for treating AUD.

## Author Contributions

RMa, EM, AL, and RMo conceived and/or designed experiments. RMa and TB performed the experiments. RMa analyzed the data. RMa and RMo interpreted the results. RMa wrote the paper. EM, TB, AL, and RMo provided critical commentary and/or revisions to the paper.

## Conflict of Interest Statement

The authors declare that the research was conducted in the absence of any commercial or financial relationships that could be construed as a potential conflict of interest.
